# Oral Fat Sensing and *CD36* Gene Polymorphism in Algerian Lean and Obese Teenagers

**DOI:** 10.3390/nu7115455

**Published:** 2015-11-04

**Authors:** Hadjer Daoudi, Jiří Plesník, Amira Sayed, Omar Šerý, Abdelkader Rouabah, Leila Rouabah, Naim Akhtar Khan

**Affiliations:** 1 Laborartoire de Biologie Cellulaire & Moléculaire, Université de Constantine 1, Constantine 25000, Alegria; hadjer25dz@hotmail.com (H.D.); mirasayed0411@gmail.com (A.S.); abdourouabah@hotmail.fr (A.R.); leilarouabah27@yahoo.fr (L.R.); 2 Physiologie de la Nutrition & Toxicologie, UMR U866 INSERM/Université de Bourgogne/Agro-Sup, Dijon 21000, France; plesnikjiri@gmail.com; 3 Laboratory of Neurobiology and Molecular Psychiatry, Department of Biochemistry, Faculty of Science, Masaryk University, Kotlářská 2, 61137 Brno, Czech Republic; 4 Laboratory of Molecular Physiology, Department of Biochemistry, Faculty of Science, Masaryk University, Kotlářská 2, 61137 Brno, Czech Republic; omarsery@sci.muni.cz; 5 Institute of Animal Physiology and Genetics, Academy of Science, Veveří 97, 60200 Brno, Czech Republic

**Keywords:** CD36, taste, obesity, adolescents, oleic acid

## Abstract

Growing number of evidences have suggested that oral fat sensing, mediated by a glycoprotein CD36 (cluster of differentiation 36), plays a significant role in the development of obesity. Indeed, a decreased expression of CD36 in some obese subjects is associated with high dietary fat intake. In the present study, we examined whether an increase in body mass index (BMI) is associated with altered oleic acid lingual detection thresholds and blood lipid profile in young Algerian teenagers (*n* = 165). The obese teenagers (*n* = 83; 14.01 ± 0.19 years; BMI *z*-score 2.67 ± 0.29) exhibited higher lingual detection threshold for oleic acid than lean participants (*n* = 82, 13.92 ± 0.23 years; BMI *z*-score 0.03 ± 0.0001). We also studied the association between rs1761667 polymorphism of *CD36* gene and obesity. The AA and AG genotypes were more frequent in obese teenagers, whereas GG genotype was more common in lean participants. The A-allele frequency was higher in obese teenagers than that in lean children. We report that rs1761667 polymorphism of *CD36* gene and oro-gustatory thresholds for fat might play a significant role in the development of obesity in young teenagers.

## 1. Introduction

During the last decades, obesity has become one of the major health issues for our civilization with its increasing prevalence in all age groups. According to WHO, there are more than 1.9 billion obese adults and 42 million overweight young children worldwide [1]. It is generally accepted that obesity is influenced by environmental and genetic factors [2]. However, one of the key factors is also an excess of fat in our diet which, associated with the lack of physical activity, leads to an increase in body mass index (BMI) [3,4]. Dietary fat provides more than twofold energy compared to proteins and carbohydrates, thus high consumption of lipids would worsen obesity and result into several pathologies like atherosclerosis, hypertension, and some other diseases [3,4].

Dietary fat is mainly perceived by its textural properties [5]. Nevertheless, growing evidences indicate the existence of another factor, *i.e*., taste for fat, which could play a role in the attraction for dietary lipids [6]. There are two main long-chain fatty acid receptors, *i.e.*, CD36 and GPR120, which play a role in the gustatory detection of lipids. The CD36 (also known as FAT, fatty acid translocase) belongs to the scavenger receptor family, and is known to bind to various ligands such as thrombospondin-1, oxidized low-density lipoproteins, growth hormone (GH)-releasing peptides and also dietary fatty acids [7]. The GPR120 belongs to the G-protein-coupled receptor (GPCR) family and is expressed in human and rodent taste bud cells [8]. Recent studies conducted on animal models and *in vitro* cell cultures showed possible alternative roles of GPR120 and CD36 in oral fat sensing. Hence, GPR120 seems to play a role in post-prandial regulation, whereas CD36 serves as a primary fat taste sensor in the lingual epithelium [6,9,10,11].

It has been previously shown that a single nucleotide polymorphism (SNP) rs1761667 of *CD36* gene, located in the 5’ flanking exon 1A area [10], is associated with the decreased expression of CD36 protein [12]. This *CD36* gene polymorphism has been associated with some pathologies like coronary artery disease [10,13] and type 2 diabetes mellitus [14]. Besides, rs1761667 polymorphism has been shown to influence gustatory perception of dietary lipids in humans. The first evidence of the impact of rs1761667 polymorphism on oral fat sensing was reported by Pepino *et al.* [15] who showed that A-allele is associated with decreased oro-gustatory detection of oleic acid in some Afro-American obese subjects. We recently conducted a study on obese Tunisian women and showed that the participants with A-allele of rs1761667 polymorphism exhibited decreased oral sensitivity (high thresholds) to oleic acid [16]. In another study conducted on young Algerian children age seven to eight, we have observed higher A-allele frequency of rs1761667 polymorphism in obese children compared to leans [17]. As expected, the obese young children exhibited higher detection threshold for oleic acid than lean participants [17]. Moreover, in the recent study Melis *et al.* [18] have shown that high expression of *CD36* (influenced by rs1761667) may by the determining factor for oral detecting of dietary fat predominantly in subjects with the low density of taste papillae.

The early period of childhood and adolescence is critical for the development of obesity in the later stage of life. It has been shown that young obese teenagers, predominantly males, are unable to return to the normal healthy state [19]. Risk factors for childhood obesity include parental fatness, social status, birth weight, timing or rate of growth, physical activity, dietary factors, and other behavioral or psychological factors [20]. Childhood obesity has been shown to result into high central adiposity and high blood pressure including high carotid extra-medial thickness in adulthood [21]. Janssen *et al*. [22] have clearly shown that overweight and obesity during childhood are strong predictors of obesity and risk for coronary heart disease in young adults. Longitudinal studies have demonstrated that the transition from childhood to adulthood should be taken into account to build obesity prediction models [23]. Hence, it seems imperative to know better the predictive factors of childhood obesity to avoid the obesity-associated complications in adulthood.

As mentioned above, there seems a relationship between decreased oral fat sensing and *CD36* SNP in adult and young obese subjects; however, no such study is available in teenagers. We, therefore, conducted the present study to investigate the relationship between rs1761667 polymorphism of *CD36* gene, oral fatty acid detection thresholds in young lean and obese Algerian teenagers.

## 2. Experimental Section

### 2.1. Subjects

We recruited (*n* = 165) male and female adolescents from Constantine district in Algeria. All the participants belonged to Arab-Berber ethnicity. The study was conducted on a young population (Table 1). The exclusion criteria for participants were any history of a chronic pathology such as cardiovascular disease, diabetes, liver, or kidney disease. The smokers were also excluded from the study. A written consent was obtained from all participants and their parents, and they were assured about the confidentiality of the study. All personal data, such as names and dates of birth, were erased from the database.

**Table 1:** Characteristics of study groups and concentrations of blood parameters between controls and obese participants.

Parameters	Control Participants (*n* = 82)	Obese Participants (*n* = 83)
Age (years)	13.92 ± 0.23	14.01 ± 0.19
BMI *z*-score	0.03 ± 0.00	2.67 ± 0.29 **
Glycemia (mmol/L)	4.41 ± 0.06	4.76 ± 0.05 *
TC (mmol/L)	3.04 ± 0.08	3.39 ± 0.07 *
LDL-C (mmol/L)	1.64 ± 0.07	2.00 ± 0.06 **
HDL-C (mmol/L)	1.08 ± 0.03	0.91 ± 0.02 **
TG (mmol/L)	0.74 ± 0.04	1.04 ± 0.05 **
Insulin (pmol/L)	45.98 ± 0.69	54.38 ± 2.22 **
HOMA index	1.29 ± 0.03	1.70 ± 0.12 **

* *p* < 0.05, ** *p* < 0.01 between controls and obese. Abbreviations: TC (total cholesterol); LDL-C (low-density lipoprotein cholesterol); HDL-C (high-density lipoprotein cholesterol); TG (triglycerides); HOMA (homeostasis model assessment).

#### 2.1.1. *Ethics*

The study was carried out in accordance with the Declaration of Helsinki (1989) of the World Medical Association, and the research council of the University of Constantine-1 approved the study protocol (10 September 2014). Our experimental protocol conforms to the relevant ethical guidelines for human research.

### 2.2. BMI z-Score

The BMI of teenagers was calculated as per WHO guidelines and expressed as *z*-score [24]. The lean subjects had a BMI *z*-score below 1 and obese more than 2. To observe a clear difference between lean and obese groups, the subjects with BMI *z*-score between 1 and 2 were excluded from the study.

### 2.3. Determination of Fasting Blood Glucose and Lipids Parameters

Venous blood from all the subjects was collected in heparinized tubes. The concentrations of fasting glucose, total cholesterol (TC), and triglycerides (TG) were determined by Biochemical analyzer XL 200 (ErbaLachema, Mannheim, Germany). LDL and HDL cholesterol levels were measured by cholesterol oxidase method (BioSystems, Barcelona, Spain). Insulin concentrations were determined by ELISA (RayBio, Norcross, GA, USA).

### 2.4. Oleic Acid Sensitivity Analysis

The participants were called on a stipulated date and advised to come early in the morning without taking breakfast (fasting state). The subjects were weighed and a blood sample was drawn, before the sensitivity test, to assess blood parameters. We used the alternative-forced choice (AFC) method as described before [16,17]. Briefly, different concentrations of oleic acid, OA (0.018, 0.18, 0.37, 0.75, 1.5, 3, 6, and 12 mmol/L) were prepared and the teenagers were subjected to taste, one-by-one, the three solutions. One solution contained OA with acacia gum (0.01%) and the other two served as controls with 0.01% acacia gum only. The taste sessions were performed in an isolated chamber, close to the laboratory. Control samples were prepared in the same way but without added oil. We started with the lowest OA concentration, and the detection threshold was established when the subject identified twice the same solution containing OA. The participants were asked to use a nose clip to minimalize olfaction cues during the test and to rinse the mouth between every tasting. The teenagers were not allowed to drink the solutions, rather they had to spit them out after keeping the solution in mouth for few seconds.

### 2.5. Genotyping Analysis

Genomic DNA (gDNA) was extracted from venous blood, using Wizard^®^ Genomic DNA Purification Kit (Promega, USA). Rs1761667 polymorphism of CD36 gene was genotyped using PCR-RFLP. As per our method [16,17], the gDNA was amplified with Kapa mix, containing Taq polymerase (Kapa Biosystems, Wilmington, MA, USA) with forward and reverse primers (5’-CAA AAT CAC AAT CTA TTC AAG ACCA-3’ and 5’-TTT TGG GAG AAA TTC TGA AGA G-3’). After amplification, the 190 bp PCR product was digested by *HhaI* endonuclease (Thermo Fisher Scientific, Waltham, MA, USA) which cleaves the product into two fragments of 138 bp and 52 bp if the G-allele is present, whereas in the presence of A-allele we observed undigested 190 bp product. The final products were separated and analyzed in 2% agarose gel electrophoresis, stained with ethidium bromide.

### 2.6. Statistical Analysis

Statistical analysis was conducted by Statistica 14 software (Statsoft, Tulsa, OK, USA). One-way ANOVA was used to compare the difference between parameters in the study groups. For correlation between various parameters, Spearman rank correlation was performed. Hardy-Weinberg equilibrium (HWE) was assessed by chi-square (χ^2^) test. For the comparison of allelic and genotype frequencies between obese and control, Fisher exact test was used. All data in the tables and figures are presented as means ± SEM, and *p* < 0.05 was considered as statistically significant.

## 3. Results

### 3.1. Characteristics of the Participants

The teenager participants (*n* = 165) were divided into two groups: obese with a BMI *z*-score higher than 2 (*n* = 83 (females = 39, males = 44), *z*-score 2.67 ± 0.29) and leans with a BMI *z*-score below 1 (*n* = 82 (females = 37, males = 45), *z*-score 0.03 ± 0.0). The average age of the subjects was 13.9 ± 1.1 years.

### 3.2. Blood Parameters

Table 1 shows that both lean and obese young teenagers had fasting glucose concentrations within normal range, though the latter had slightly higher glycemia than the former (*p* < 0.05). Similarly, total cholesterol (TC) concentration was normal in both the groups, but obese participants had higher TC concentration than control children (*p* < 0.05). Lean participants had higher HDL-C concentration compared to obese teenagers (*p* < 0.01). Obese children had significantly elevated LDL-C concentration compared to lean ones (*p* < 0.01). Triglycerides (TG) concentration was higher in obese teenagers than that in lean participants (*p* < 0.01). Insulin concentration was also higher in obese teenagers than that in lean children (*p* < 0.01). We observed a positive association between total TC and TG, HDL-C and LDL-C levels (*p* < 0.01, *p* < 0.04, *p* < 0.01 respectively). TG concentration was positively correlated with LDL-C (*p* < 0.01) and negatively with HDL-C levels (*p* < 0.01). Fasting glucose concentration was positively correlated with insulin level (*p* = 0.026). HOMA index was also higher in obese participants that that in lean ones. No difference between boys and girls was observed as regards the above-mentioned parameters.

### 3.3. Oleic Acid Sensitivity

**Figure 1:**
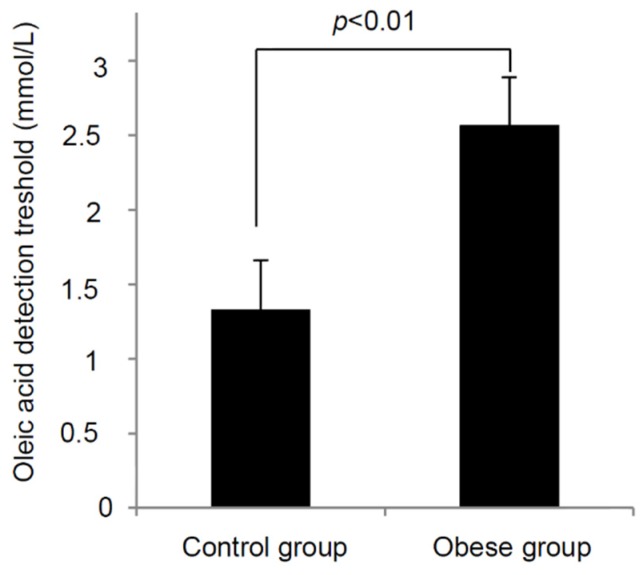
Relationship between BMI and oro-sensory detection of a fatty acid in young leans and obese children. The oleic acid detection thresholds were determined in lean (*n* = 82) and obese children (*n* = 83) as described in the Materials and Methods section. The results are means ± SEM.

**Figure 2:**
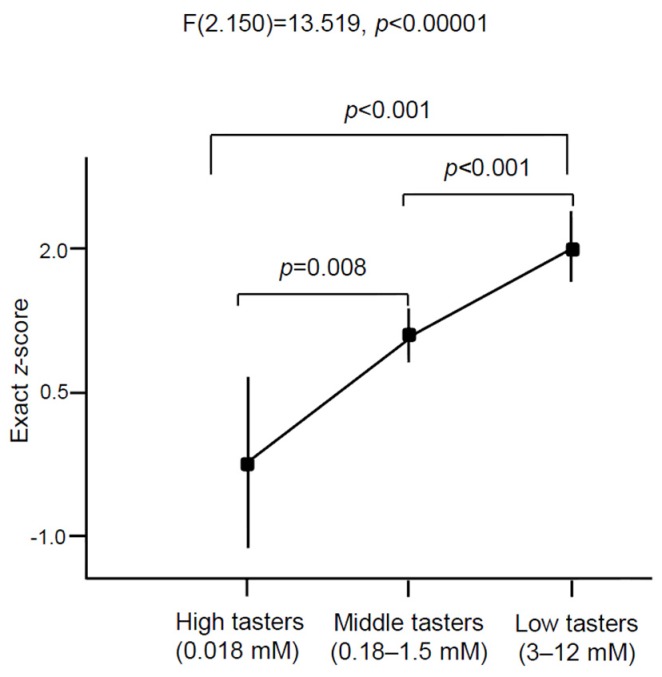
Fatty acid sensitivity in all young teenagers in relation to BMI. The lean and obese children (*n* = 165) were divided into three groups on the basis of oro-sensory detection of oleic acid as high, middle, and low tasters. “High tasters” group contained lean teenagers only (*n* = 8), most of the teenagers from the both groups (control, *n* = 60; obese, *n* = 45) belonged to the “Middle tasters” group and the “Low tasters” group consisted predominantly of obese participants (*n* = 41) and controls (*n* = 11). The results are means ± SEM.

We observed statistically significant difference in oleic acid oral detection threshold between obese and lean adolescents (Figure 1). Obese subjects exhibited almost twofold OA detection threshold (2.57 ± 0.29 mmol/L, *p* < 0.01) than lean participants (1.33 ± 0.15 mmol/L). We noticed a positive correlation between BMI *z*-score and OA detection (*p* < 0.01). If we divide all the participants, on the basis of oral detection thresholds, into three categories: high tasters (between 0 to 0.018 mM), middle tasters (between 0.18 and 1.5 mM), and low tasters (between 3 and 12 mM), we notice a relationship between BMI and fat taste thresholds (*p* < 0.001; Figure 2). We did not find any significant difference in the measured parameters between genders.

### 3.4. CD36 Genotyping

**Figure 3:**
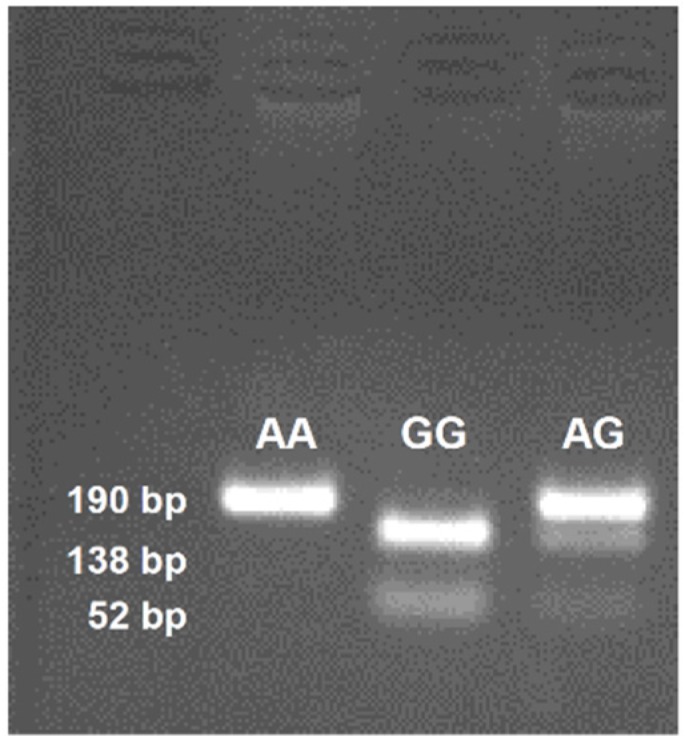
Rs1761667 genotypes separated on 2% agarose gel and stained with ethidium bromide. The blot shows one identical photograph from several reproduced ones.

Figure 3 shows rs1761667 genotypes on agarose gel. We did not observe any deviation from Hardy-Weinberg equilibrium (*p* > 0.05) in lean and obese participants (Table 2) in genotype frequencies of rs1761667 polymorphism of *CD36* gene. The frequencies of A-allele in lean and obese groups were 56.7% and 68.1%, respectively (*p* = 0.041, OR = 1.63; 95% CI of OR = 1.04–2.55). AA and AG genotypes are present predominantly in obese teenagers (*p* = 0.008; *p* = 0.002, respectively). Minor genotype was, on the other hand, present in the controls. We did not find any significant difference between CD36 genotype and oleic acid oral sensitivity threshold. Similarly, we did not observe any significant difference between CD36 genotypes and BMI *z*-score neither in obese nor control teenagers (*p* = 0.58; *p* = 0.41, respectively). We did not find any significant difference between the genders.

**Table 2:** Genotype and allelic frequencies of *CD36* rs1761667 between control and obese participants.

Parameters	Control Participants (*n* = 82)	Obese Participants (*n* = 83)	Statistical Calculations
HWE χ^2^	2.67	3.05	NS
Alleles (%)			*p* = 0.041
A	93 (56.7)	113 (68.1)	OR = 1.63; 95% CI
G	71 (43.3)	53 (31.9)	RR = 1.28; 95% CI
Genotypes (%)			
AA	30 (36.6)	35 (42.2)	*p* = 0.008
AG	33 (40.2)	43 (51.8)	*p* = 0.002
GG	19 (23.2)	5 (6.0)	-

**Abbreviations**: HWE χ^2^ (Hardy-Weinberg equilibrium χ^2^); MAF (Minor allele frequency); OR (odd ratio); RR (relative risk).

## 4. Discussion

It has been previously shown that the subjects which are obese at a young age became severely obese in adulthood [25,26]. Excess of caloric intake, largely contributed by fat overconsumption, seems to be one of the factors implicated in this pathology [6]. Moreover, altered oro-gustatory perception of lipids has been associated with obesity [6]. It, therefore, seems mandatory to shed light on oral fat sensing that might take part in the regulation of feeding behavior in obese subjects.

As regards blood parameters, we observed higher glycemia, LDL-C, triglycerides, and insulin concentrations in obese children than the lean participants. It has been previously shown that the teenagers with a high degree of obesity exhibited high blood concentrations of LDL-C, glucose and insulin [27]. Similar results were also obtained in an American population, where the prevalence of hyperinsulinemia and hypertriglyceridemia was significantly higher in severely obese children and adolescents, compared to the less obese individuals [28]. We noticed low HDL-C concentrations in the obese group. Indeed, Ruel *et al*. [29] have reported that low HDL is associated with high BMI and waist circumference. Jiang *et al*. [30] have also shown that insulin levels were positively correlated with serum triglyceride, and negatively with HDL-C levels in all age group obese children including 12–17 years old participants. These investigators concluded that these changes in obese children might have adverse consequences for cardiovascular diseases in adulthood. Furthermore, in obese children, we also observed a high HOMA index, an indicator of insulin-resistance (IR) which is directly associated with the aggravation of obesity [31].

As regards the gustatory detection of lipids, we noticed that obese participants exhibited a significantly higher detection threshold (lower sensitivity) compared to lean participants. Whilst the “High tasters” group is composed mainly of controls, in “Low tasters” group we can find predominantly obese teenagers. Previous studies performed on Australian [32] and Tunisian [16] adults also showed that the obese subjects exhibited reduced oleic acid sensitivity. Low fatty acid oro-sensory detection in obesity has been attributed to low expression of CD36 protein in the mouse [33] or to AA genotype of rs1761667 polymorphism of CD36 in human beings [12,16,17]. However, we did not observe a relationship between CD36 gene AA genotype and fatty acid detection thresholds. The reason for this failure might be the less developed papillae which might not have expressed sufficiently the truncated CD36 protein, transduced by CD36 rs1761667 AA genotype [12], in the young Algerian children. In fact, it has been shown that fungiform papillae attain full size at the age of 8–10 years, and the circumvallate papillae, located in the posterior region, continue to grow until the age of 15–16 years [34]. This argument is pertinent as the circumvallate papillae have been shown to express nine-time higher CD36 mRNA than fungiform papillae [35]. Alternatively, it is also possible that a variant of GPR120, another lipido-receptor that is associated with obesity in a European study [36], might be involved in low oro-sensory sensitivity in obese Algerian teenagers; however, further studies are required to confirm this hypothesis. We also noted higher A-allele frequency compared to G-allele in our study, and this kind of distribution has been, so far, reported in Arabic populations, namely in Tunisia [16] and Algeria [17]. Interestingly, previous studies conducted on different populations, namely Caucasians [18], Indians [14], and Asians [13] showed a high frequency of G-allele.

Nonetheless, A-allele frequency of rs1761667 polymorphism of CD36 gene was higher in obese children than lean participants. A-allele was found to be associated with the intake of soda and French fries in obese children, suggesting that fat-containing products might influence, in the long-term, the fatty acid oro-sensory detection capacity. Our hypothesis is supported by the observations of Stewart *et al*. [37] who have reported that feeding a high-fat diet significantly increased oleic acid oral detection threshold in lean subjects. Similarly, feeding a high-fat diet in mice resulted in high oro-sensory threshold for linoleic acid [11].

Ours is the first study to show an association between high oro-sensory threshold for a fatty acid and obesity in 13–14 year old teenagers. These results might be confirmed in other young population with different cultural and eating habits. Though the obese participants had CD36 A-allele, it was not associated with high oro-detection threshold for the fatty acid. Besides, we cannot rule out an influence of altered levels of sex hormones in obese teenagers on fat taste perception and other parameters. It is also difficult to determine whether oral fat perception sensitivity affects fat intake or body weight regulation. Future studies are required to address these questions.

## References

[1] George A.M., Jacob A.G., Fogelfeld L. (2015). Lean diabetes mellitus: An emerging entity in the era of obesity. World J. Diabetes.

[2] Ang Y.N., Wee B.S., Poh B.K., Ismail M.N. (2012). Multifactorial Influences of Childhood Obesity. Curr. Obes. Rep..

[3] Barnett T.A., Maximova K., Sabiston C.M., van Hulst A., Brunet J., Castonguay A.L., Bélanger M., O’Loughlin J. (2013). Physical activity growth curves relate to adiposity in adolescents. Ann. Epidemiol..

[4] Paradis A.M., Godin G., Pérusse L., Vohl M.C. (2009). Associations between dietary patterns and obesity phenotypes. Int. J. Obes..

[5] Rolls E.T. (2015). Taste, olfactory, and food reward value processing in the brain. Prog. Neurobiol..

[6] Gilbertson T.A., Khan N.A. (2014). Cell signaling mechanisms of oro-gustatory detection of dietary fat: Advances and challenges. Prog. Lipid. Res..

[7] Silverstein R.L., Febbraio M. (2010). CD36, a scavenger receptor involved in immunity, metabolism, angiogenesis, and behavior. Sci. Signal..

[8] Cartoni C., Yasumatsu K., Ohkuri T., Shigemura N., Yoshida R., Godinot N., le Coutre J., Ninomiya Y., Damak S. (2010). Taste preference for fatty acids is mediated by GPR40 and GPR120. J. Neurosci..

[9] Ancel D., Bernard A., Subramaniam S., Hirasawa A., Tsujimoto G., Hashimoto T., Passilly-Degrace P., Khan N.A., Besnard P. (2015). The oral lipid sensor GPR120 is not indispensable for the orosensory detection of dietary lipids in the mouse. J. Lipid Res..

[10] Ma X., Bacci S., Mlynarski W., Gottardo L., Soccio T., Menzaghi C., Iori E., Lager R.A., Shroff A.R., Gervino E.V. (2004). A common haplotype at the CD36 locus is associated with high free fatty acid levels and increased cardiovascular risk in Caucasians. Hum. Mol. Genet..

[11] Ozdener M.H., Subramaniam S., Sundaresan S., Sery O., Hashimoto T., Asakawa Y., Besnard P., Abumrad N.A., Khan N.A. (2014). CD36- and GPR120-mediated Ca^2+^ signaling in human taste bud cells mediates differential responses to fatty acids and is altered in obese mice. Gastroenterology.

[12] Love-Gregory L., Sherva R., Schappe T., Qi J.S., McCrea J., Klein S., Connelly M.A., Abumrad N.A. (2011). Common CD36 SNPs reduce protein expression and may contribute to a protective atherogenic profile. Hum. Mol. Genet..

[13] Zhang Y., Ling Z.Y., Deng S.B., Du H.A., Yin Y.H., Yuan J., She Q., Chen Y.Q. (2014). Associations between CD36 gene polymorphisms and susceptibility to coronary artery heart disease. Braz. J. Med. Biol. Res..

[14] Banerjee M., Gautam S., Saxena M., Bidb H.K., Agrawalc C.G. (2010). Association of CD36 gene variants rs1761667 (G > A) and rs1527483 (C > T) with Type 2 diabetes in North Indian population. Int. J. Diabetes. Mellit..

[15] Pepino M.Y., Love-Gregory L., Klein S., Abumrad N.A. (2012). The fatty acid translocase gene CD36 and lingual lipase influence oral sensitivity to fat in obese subjects. J. Lipid Res..

[16] Mrizak I., Šerý O., Plesnik J., Arfa A., Fekih M., Bouslema A., Zaouali M., Tabka Z., Khan N.A. (2015). The A allele of cluster of differentiation 36 (CD36) SNP 1761667 associates with decreased lipid taste perception in obese Tunisian women. Br. J. Nutr..

[17] Sayed A., Šerý O., Plesnik J., Daoudi H., Rouabah A., Rouabah L., Khan N.A. (2015). CD36 AA genotype is associated with decreased lipid taste perception in young obese, but not lean, children. Int. J. Obes..

[18] Melis M., Sollai G., Muroni P., Crnjar R., Barbarossa I.T. (2015). Associations between orosensory perception of oleic acid, the common single nucleotide polymorphisms (rs1761667 and rs1527483) in the CD36 gene, and 6-*n*-propylthiouracil (PROP) tasting. Nutrients.

[19] Dietz H. (1994). Critical periods in childhood for the development of obesity. Am. J. Clin. Nutr..

[20] Parsons T.J., Power C., Logan S., Summerbell C.D. (1999). Childhood predictors of adult obesity: A systematic review. Int. J. Obes. Relat. Metab. Disord..

[21] Skilton M.R., Marks G.B., Ayer J.G., Garden F.L., Garnett S.P., Harmer J.A., Leeder S.R., Toelle B.G., Webb K., Baur L.A. (2013). Weight gain in infancy and vascular risk factors in later childhood. Pediatrics.

[22] Janssen I., Katzmarzyk P.T., Srinivasan S.R., Chen W., Malina R.M., Bouchard C., Berenson G.S. (2005). Utility of childhood BMI in the prediction of adulthood disease: Comparison of national and international references. Obes. Res..

[23] Tremblay L., Rinaldi C.M. (2010). The prediction of preschool children’s weight from family environment factors: Gender-linked differences. Eat. Behav..

[24] Must A., Anderson S.E. (2006). Body mass index in children and adolescents: considerations for population-based applications. Int. J. Obes..

[25] Hughes A.R., Sherriff A., Lawlor D.A., Ness A.R., Reilly J.J. (2011). Incidence of obesity during childhood and adolescence in a large contemporary cohort. Prev. Med..

[26] Natalie S., Suchindran C., North K.E., Popkin B.M., Gordon-Larsen P. (2010). Association of adolescent obesity with risk of severe obesity in adulthood. JAMA.

[27] Lavrador M.S., Abbes P.T., Escrivão M.A., Taddei J.A. (2011). Cardiovascular risks in adolescents with different degrees of obesity. Arq. Bras. Cardiol..

[28] Weiss R., Dziura J., Burgert T.S., Tamborlane W.V., Taksali S.E., Yeckel C.W., Allen K., Lopes M., Savoye M., Morrison J. (2004). Obesity and the metabolic syndrome in children and adolescents. N. Engl. J. Med..

[29] Ruel I.L., Gaudet D., Perron P., Bergeron J., Julien P., Lamarche B., Québec LipD Study (2003). Effect of obesity on HDL and LDL particle sizes in carriers of the null P207L or defective D9N mutation in the lipoprotein lipase gene: The Québec LipD Study. Int. J. Obes. Relat. Metab. Disord..

[30] Jiang X., Srinivasan S., Webber L., Wattigney W.A., Berenson G.S. (1995). Association of fasting insulin level with serum lipid and lipoprotein levels in children, adolescents, and young adults: The Bogalusa Heart Study. Arch. Intern. Med..

[31] Lee J.M., Okumura M.J., Davis M.M., Herman W.H., Gurney J.G. (2006). Prevalence and determinants of insulin resistance among U.S. adolescents: A population-based study. Diabetes Care..

[32] Stewart J.E., Seimon R.V., Keast R.S., Clifton P.M., Feinle-Bisset C. (2011). Marked differences in gustatory and gastrointestinal sensitivity to oleic acid between lean and obese men. Am. J. Clin. Nutr..

[33] Chevrot M., Bernard A., Ancel D., Buttet M., Martin C., Abdoul-Azize S., Merlin J.F., Poirier H., Niot I., Khan N.A. (2013). Obesity alters the gustatory perception of lipids in the mouse: Plausible involvement of lingual CD36. J. Lipid Res..

[34] Temple E.C., Hutchinson I., Laing D.G., Jinks A.L. (2002). Taste development: Differential growth rates of tongue regions in humans. Brain Res. Dev. Brain Res..

[35] Laugerette F., Passilly-Degrace P., Patris B., Niot I., Febbraio M., Montmayeur J.P., Besnard P. (2005). CD36 involvement in orosensory detection of dietary lipids, spontaneous fat preference, and digestive secretions. J. Clin. Invest..

[36] Ichimura A., Hirasawa A., Poulain-Godefroy O., Bonnefond A., Hara T., Yengo L., Kimura I., Leloire A., Liu N., Iida K. (2012). Dysfunction of lipid sensor GPR120 leads to obesity in both mouse and human. Nature.

[37] Stewart J.E., Keast R.S. (2012). Recent fat intake modulates fat taste sensitivity in lean and overweight subjects. Int. J. Obes..

